# Recombinant thrombomodulin alleviates oxidative stress without compromising host resistance to infection in rats infected with methicillin-resistant *Staphylococcus aureus*

**DOI:** 10.1038/s41598-020-74529-4

**Published:** 2020-10-15

**Authors:** Takashi Ito, Binita Shrestha, Yasuyuki Kakihana, Ikuro Maruyama

**Affiliations:** 1 Department of Systems Biology in Thromboregulation, Kagoshima University Graduate School of Medical and Dental Sciences, 8-35-1 Sakuragaoka, Kagoshima, 890-8544 Japan; 2 Department of Emergency and Intensive Care Medicine, Kagoshima University Graduate School of Medical and Dental Sciences, Kagoshima, Japan; 3 Department of Medicinal Chemistry, Rogel Cancer Center, College of Pharmacy, University of Michigan, Ann Arbor, MI USA

**Keywords:** Infection, Coagulation system

## Abstract

Recombinant thrombomodulin (rTM) has been used for treatment of sepsis-associated disseminated intravascular coagulation. Recent studies have suggested that anticoagulant therapy might dampen the protective role of immunothrombosis. We examined if rTM might worsen infectious diseases. Male Sprague–Dawley rats with jugular-vein catheterization were divided into three groups: no anticoagulation; rTM pretreatment; rTM treatment at 6 h. Live methicillin-resistant *Staphylococcus aureus* (MRSA) was inoculated into the tail vein of rats. rTM was administered into the jugular-vein catheter before or 6 h after MRSA inoculation, while an equal volume of saline was administered in the no-anticoagulation group. Blood samples were collected from the jugular-vein catheter before, 6 h and 12 h after MRSA inoculation. Tissue samples were collected from anesthetized rats when moribund or 18 h after MRSA inoculation. The survival rate of rats in the no-anticoagulation group, rTM pretreatment group, and rTM treatment at 6-h group was 50%, 25%, and 75%, respectively. Bacterial burden in blood, lung, liver, and spleen was neither increased nor decreased in rats treated with rTM. The ratio of bacteria found in the extravascular space to those in the intravascular space was increased in rats treated with rTM although the statistical power for this was low because of the small sample size. Metabolomics analysis revealed that rTM treatment alleviated oxidative stress, as evidenced by the decrease in levels of oxidized glutathione with reference to reduced glutathione. rTM did not promote bacterial propagation but alleviated oxidative stress in our rat model of bloodstream infection with MRSA. Further large-scale studies are needed to confirm these findings.

## Introduction

Sepsis can be defined as life-threatening organ dysfunction caused by dysregulated host responses to infection^1^. Dysregulated host responses include feedforward neutrophil accumulation, overproduction of reactive oxygen species, release of neutrophil extracellular traps (NETs), and a metabolic shift towards decreased oxidation of fatty acids^2–4^. Intravascular activation of coagulation is another important feature of the host response to infection^2,5^. Expression of tissue factor on monocytes and release of NETs from neutrophils are thought to be triggers for intravascular coagulation^5,6^. Coagulation under certain circumstances has a major physiologic role in innate immune defense against infection, a process known as “immunothrombosis”. However, uncontrolled immunothrombosis may lead to ischemic organ failure.

Several mechanisms have been proposed by which immunothrombosis promotes defense against infection^6^. First, immunothrombosis limits microbial dissemination by arresting microbes within the clot^7^. Second, thrombi form “barricades” which can limit microbial spread from one compartment to another^8,9^. Third, fibrin supports leukocytes in eliminating microbes^10^. Consequently, mice pretreated with coumadin have been shown to display increased hepatic bacterial burden and mortality after inoculation with *Yersinia enterocolitica*^11^.

Anticoagulant therapy has been considered as a treatment option for sepsis. It is not efficacious in sepsis overall, but may provide beneficial effects in a specific subpopulation with sepsis-associated disseminated intravascular coagulation (DIC)^12,13^, possibly by alleviating ischemic organ failure. Among anticoagulants, recombinant thrombomodulin (rTM) is a potential therapeutic agent for sepsis-associated DIC^14^. Although the randomized, placebo-controlled, multinational phase 3 study did not show a significant reduction in 28-day all-cause mortality in patients with sepsis-associated coagulopathy^15^, meta-analyses have suggested a potential survival benefit using this drug^16,17^. However, one might assume that treatment with rTM “dampens” the protective role of immunothrombosis and, thus, may worsen infectious diseases. To validate the plausibility of this hypothesis, we examined bacterial burden in organs and blood in the presence or absence of rTM in rats infected with methicillin-resistant *Staphylococcus aureus* (MRSA), a major bacterial species causing bloodstream infection and release of NETs ^18^. We also examined if the timing of rTM administration, before or after MRSA inoculation, affected bacterial burden.

## Methods

### Model of MRSA infection in rats

Experiments involving animals were approved by the Animal Care and Use Committee of Kagoshima University and Shin Nippon Biomedical Laboratories, and were compliant with the guideline for proper conduct of animal experiments established by Science Council of Japan.

Male Sprague–Dawley rats with jugular-vein catheterization were used for all experiments. MRSA (ATCC-43300) was incubated with agitation in Mueller–Hinton broth (Eiken Chemicals, Tokyo, Japan) for 2.5 h at 37 °C to achieve mid-log-phase growth. Then, MRSA was resuspended in saline, and 3.5 × 10^8^ colony-forming units (CFUs) of MRSA was inoculated into the tail vein of rats at 0 h and 5 h. Anticoagulation therapy was conducted in some rats, for which rTM (1 mg/kg; Asahi Kasei Pharma, Tokyo, Japan) was administered into the jugular-vein catheter immediately before (rTM pretreatment group) or 6 h after the first MRSA inoculation (rTM treatment at 6-h group). The general condition of rats was evaluated every 1 h. Rats were sacrificed when moribund or 18 h after the first MRSA inoculation (n = 8 in each group).

### Blood tests

Blood samples were collected from the jugular-vein catheter before, 6 h and 12 h after MRSA inoculation (n = 5 in each group). One portion of blood samples was used for bacteriology. Blood samples anticoagulated with ethylenediaminetetraacetic acid were used for automated counting of blood cells by an ADVIA120 system (Bayer Diagnostics, Leverkusen, Germany). Serum samples were used for measurement of levels of total bilirubin and creatinine by a clinical biochemistry analyzer BioMajesty JCA-BM6070 (JEOL, Tokyo, Japan). Citrated plasma samples were used for metabolomics analysis.

### Bacteriology and pathology

Anesthetized rats were sacrificed when moribund or 18 h after MRSA inoculation. Liver, spleen, and lung samples were homogenized in sterile phosphate-buffered saline (PBS, 1 mL/g tissue), and used for bacteriology (n = 5 in each group). The remainder of the organs were fixed in 10% neutral-buffered formalin, and used for pathology studies (n = 3 in each group). Samples of blood and tissue homogenates were serially diluted with sterile PBS, plated onto sheep blood agar plates (Nissui Pharmaceuticals, Tokyo, Japan), and incubated overnight at 37 °C before bacterial enumeration. Bacterial distribution in each organ was analyzed using Gram-stained tissue sections.

### Metabolomics analysis

Metabolomics analysis was undertaken at a service facility of LSI Medience (Tokyo, Japan). Briefly, plasma samples (200 µL) collected before and 12 h after MRSA inoculation were mixed with methanol (800 µL) and shaken for 15 min at room temperature. After centrifugation, the precipitates were dissolved with 10% acetonitrile aqueous solution (200 µL). After addition of internal standards, they were analyzed by liquid chromatography-mass spectrometry (LC–MS) and capillary electrophoresis-mass spectrometry (CE-MS). Tuning and calibration were carried out with standard solutions provided by Agilent Technologies (Palo Alto, CA, USA).

LC–MS was done using a LC system (HP1200; Agilent Technologies) equipped with a C18 column (2 μm, 50 mm × 2.0 mm ID, CAPCELL PAK C18 IF; Shiseido, Tokyo, Japan) coupled with an electrospray ionization quadrupole time-of-flight mass spectrometer (6520; Agilent Technologies). Solvent A was composed of 5 mM ammonium acetate aqueous solution, whereas solvent B was acetonitrile. Metabolites were eluted at a flow rate of 0.2 mL/min at 40 °C with a linear gradient of 10–90% solvent B over 10 min, followed by a further 5-min hold at 100% solvent B. The mass spectrometer was operated in positive and negative scan modes (*m/z* 60 to 1,200) with a capillary voltage of 3,500 V. The nebulizing gas pressure was 40 psi and the dry gas flow was 8 L/min at 350 °C.

Ionic metabolites were measured in the positive mode of a CE-time-of-flight mass spectrometer (6520; Agilent Technologies). Metabolites were separated in a fused-silica capillary (50 μm i.d. × 100 cm total length; GL Science, Tokyo, Japan) filled with 1 mol/L of formic acid aqueous solution (cation mode), or 20 mM ammonium formate and 20 mM ammonium acetate aqueous solution (pH 10, anion mode) as the electrolyte. The sample solution was injected at 5 kPa for 15 s (~ 15 nL), and a voltage of 30 kV was applied. The capillary tray and sample tray were maintained at room temperature and 5 °C, respectively. The sheath liquid was methanol/water (50% *v/v*) containing 5 mM ammonium acetate. The CE-time of flight mass spectrometer was operated in positive and negative scan modes (*m/z* 60 to 1,200). The capillary voltage was set at 3500 V and the nitrogen gas (heater temperature = 250 °C) flow rate was set at 10 L/min.

A data file of MS was converted to csv format with csv convertor (Agilent Technologies). All peak positions (retention time and *m/z*) and areas were calculated by Markeranalysis (LSI Medience). All peak areas were aligned into one datasheet and the errors of peak intensities were corrected by internal standards. Noise peaks were deleted compared with the peaks detected in blank samples. Metabolites were identified by comparing the retention time and *m/z* with the standard data set established by LSI Medience. Mean fold-change and the Student’s *t*-test were carried out for all detected peaks.

### Statistical analyses

Statistical analyses were carried out using SPSS v26 (IBM. Armonk NY, USA). For comparison of multiple groups over time, repeated measures analysis of variance (ANOVA) models were used. For comparison of paired two samples, Wilcoxon signed-rank test was used. For comparison of independent samples, Wilcoxon rank sum test was used. *P* < 0.05 was considered significant.

### Ethics approval and consent to participate

Experiments involving animals were approved by the Animal Care and Use Committee of Kagoshima University and Shin Nippon Biomedical Laboratories, which are both in Kagoshima, Japan.

## Results

**Figure 1:**
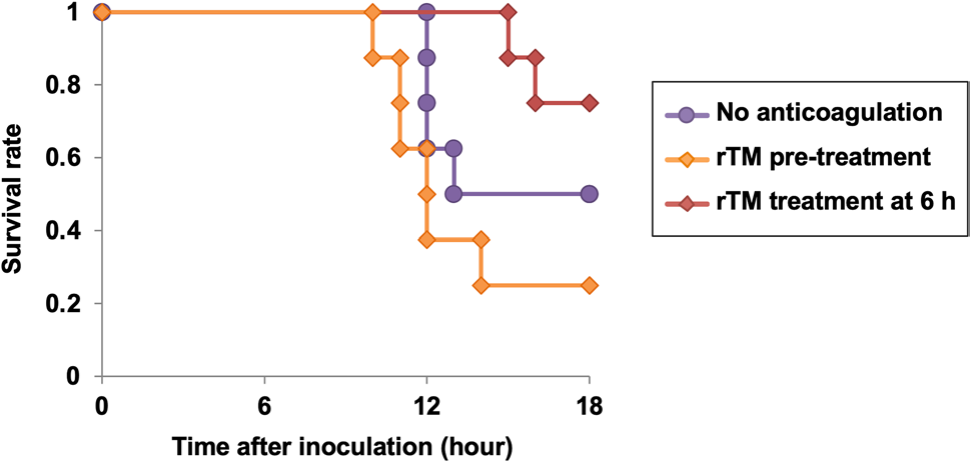
Survival benefit of treatment with recombinant thrombomodulin in MRSA-infected rats. MRSA (3.5 × 10^8^ CFU) was inoculated into the tail vein of rats at 0 h and 5 h. rTM (1 mg/kg) was administered into a jugular-vein catheter immediately before (rTM pretreatment group) or 6 h after the first MRSA inoculation (rTM treatment at 6-h group) while an equal volume of saline was administered in the no-anticoagulation group. The general condition of rats was evaluated every 1 h. Rats were sacrificed when moribund or 18 h after the first MRSA inoculation (n = 8 in each group). Survival rates up to 18 h are shown.

To test whether anticoagulation with rTM could worsen infectious diseases, we first analyzed the survival rates of MRSA-infected rats in the presence or absence of rTM administration. The survival rate of rats without rTM administration was 50% at the study endpoint (Fig. 1). The survival rate of rats treated with rTM immediately before or 6 h after MRSA inoculation was 25% and 75%, respectively. Hence, the timing of rTM administration might be an important determinant of treatment efficacy.

**Figure 2:**
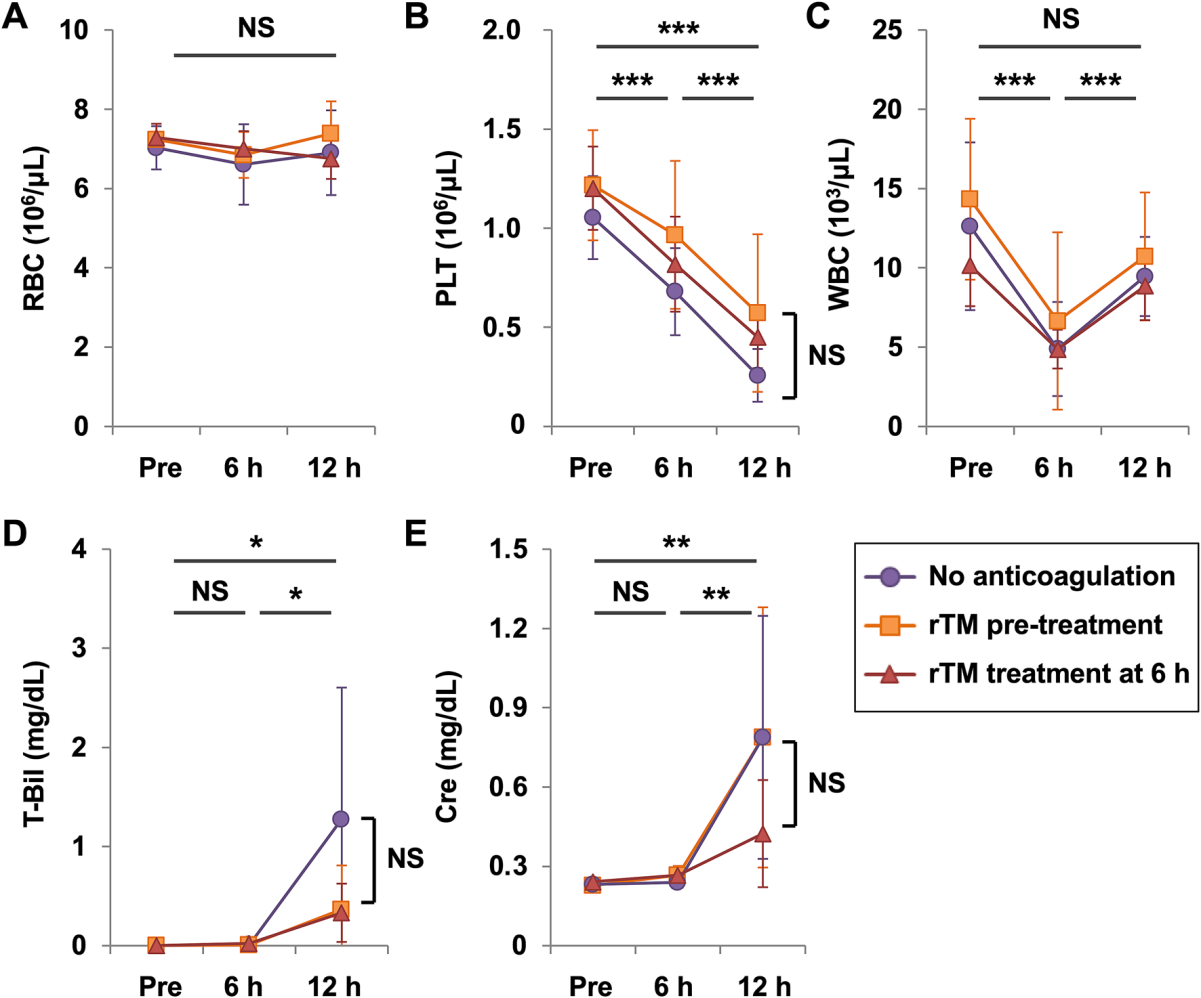
Thrombocytopenia and organ dysfunction in MRSA-infected rats. Blood samples were collected from a jugular-vein catheter before, 6 h and 12 h after MRSA inoculation (n = 5 in each group). **(A–C)** Red blood cell (RBC), platelet (PLT), white blood cell (WBC) counts in the no-anticoagulation group, rTM pretreatment group, and rTM treatment at 6-h group are shown. **(D)** Serum level of total bilirubin and **(E)** serum level of creatinine in the no-anticoagulation group, rTM pretreatment group, and rTM treatment at 6-h group are shown. Data are shown as mean ± SD. Repeated measures ANOVA models were used for comparison of three groups over time. ***P < 0.05 in all three groups. *P < 0.05 only in the no-anticoagulation group. **P < 0.05 in the no-anticoagulation group and the rTM pretreatment group. NS: not significant.

**Figure 3:**
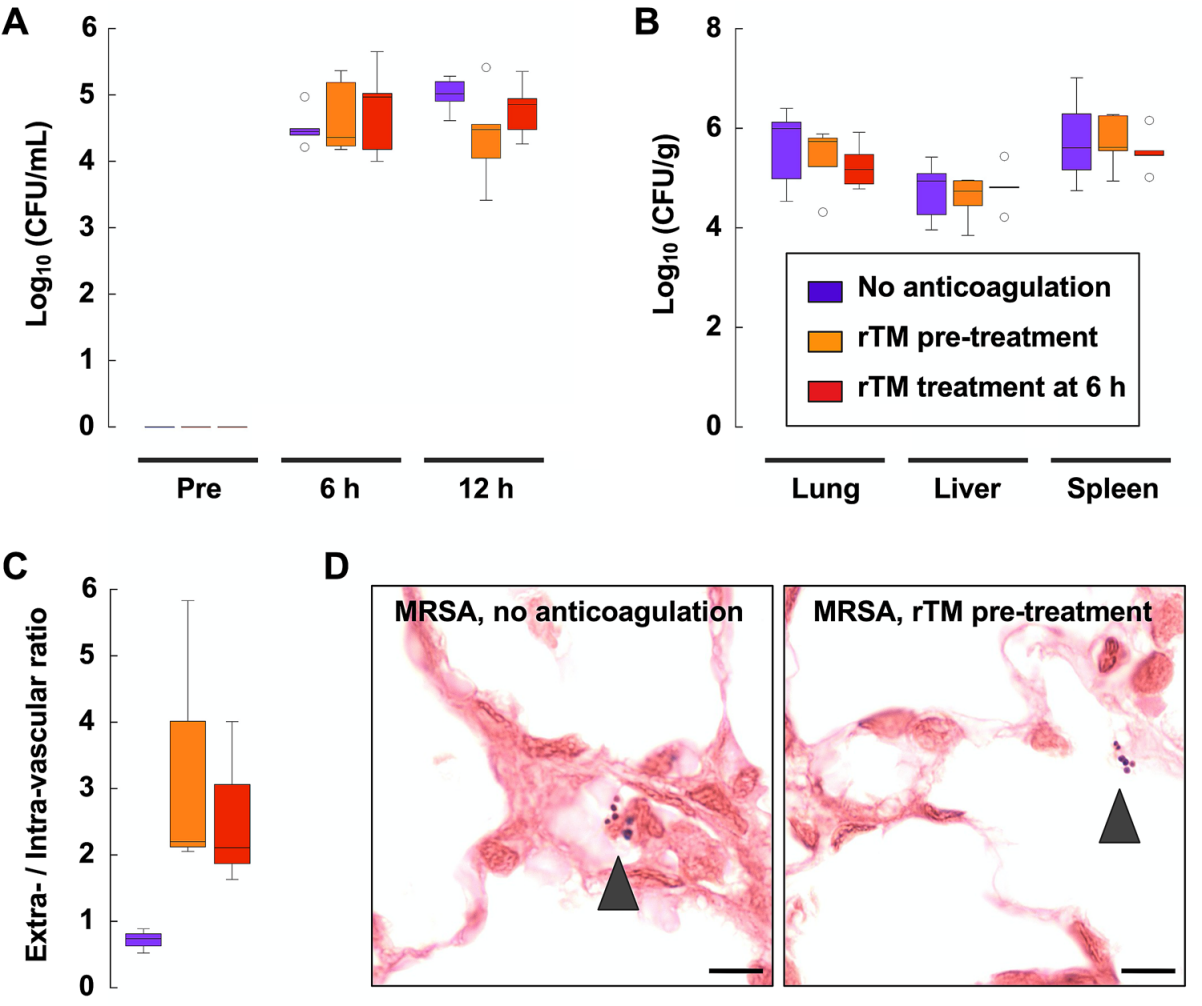
Bacterial burden in MRSA-infected rats treated with recombinant thrombomodulin. **(A)** Blood samples collected before, 6 h and 12 h after MRSA inoculation were serially diluted, plated onto sheep blood agar plates, and incubated overnight at 37 °C before bacterial enumeration. CFUs of blood samples in the no-anticoagulation group, rTM pretreatment group, and rTM treatment at 6-h group are shown in a logarithmic scale (n = 5 in each group). **(B)** CFUs of tissue homogenates of lung, liver, and spleen are shown in a logarithmic scale (n = 5 in each group). **(C) **MRSA distribution in the lung was analyzed using Gram-stained tissue sections. The number of MRSA in the extravascular and intravascular space was counted separately, and the ratio is shown (n = 3 in each group). **(D)** Representative images of Gram-stained lung tissue sections in the no-anticoagulation group and rTM pretreatment group are shown. Arrowheads show Gram-positive MRSA. Scale bar = 10 µm. Box plots show the median, lower and upper quartiles, lower and upper extreme values, and outliers.

Next, we conducted hematology and bacteriology studies to ascertain the effects of rTM administration on MRSA-induced disease. There is little change in red blood cell counts with this MRSA infection model (Fig. 2A). Thrombocytopenia and leukopenia became evident 6 h after MRSA inoculation regardless of treatment (Fig. 2B,C). Liver dysfunction and kidney dysfunction, as evidenced by an increase in the level of total bilirubin and creatinine, respectively, became prominent 12 h after MRSA inoculation in rats without anticoagulation (Fig. 2D,E). Elevation of total bilirubin and creatinine was less prominent in rats treated with rTM, though the difference was not significant when compared with those without anticoagulation (Fig. 2D,E). The bacterial burden in samples of blood, lung, liver, and spleen was neither increased nor decreased in rats treated with rTM (Fig. 3A,B). However, the ratio of bacteria found in the extravascular space to those found in the intravascular space was increased in rats treated with rTM (Fig. 3C,D). These findings suggested that rTM treatment could allow bacterial passage from the intravascular space to the extravascular space, but bacterial burden in the whole organ may be only slightly affected by rTM treatment.

**Figure 4:**
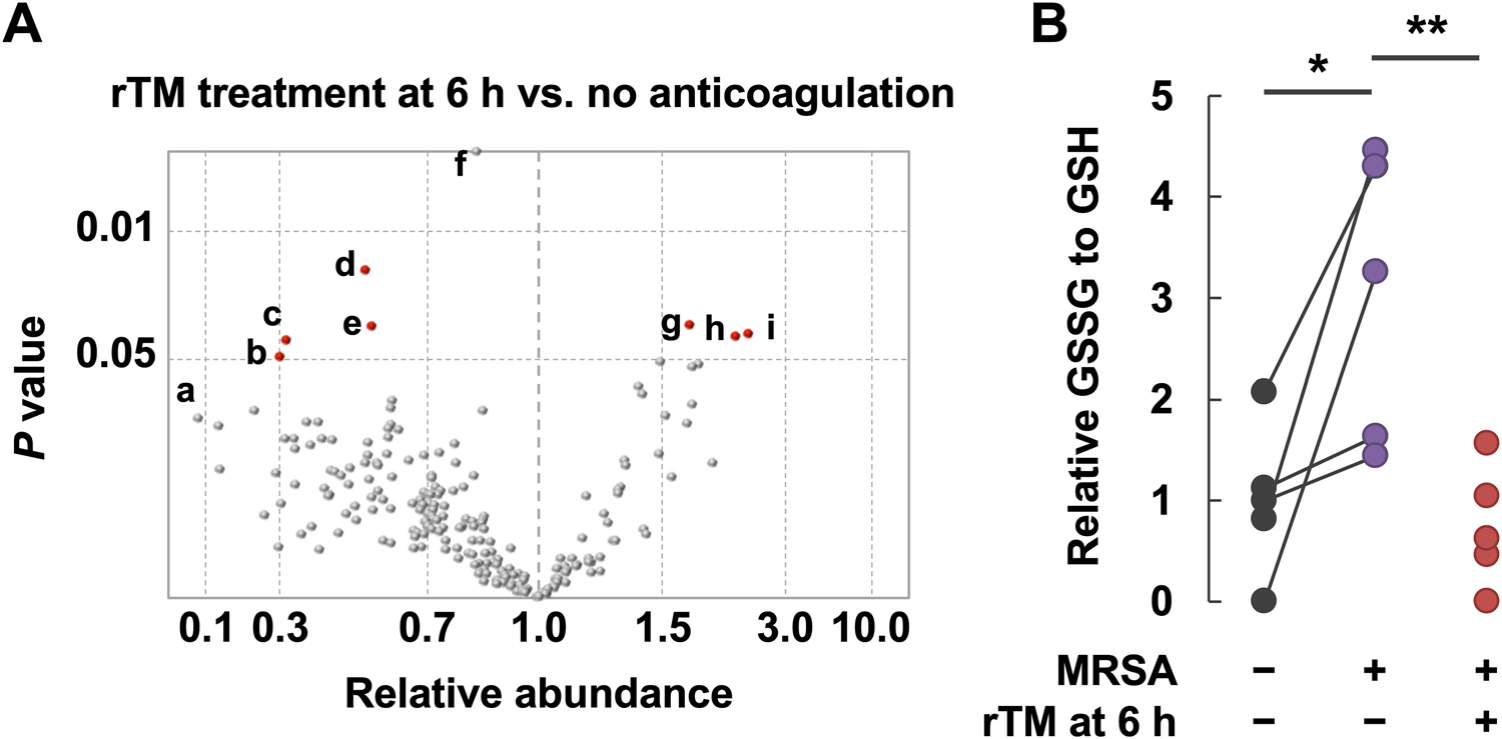
Metabolomics analysis suggests an antioxidative action of recombinant thrombomodulin in MRSA-infected rats. **(A)** Citrated plasma samples collected before and 12 h after MRSA inoculation were used for metabolomics analysis. Relative abundance and the *P*-value of each metabolite in the rTM treatment at 6-h group (n = 5) with reference to the no-anticoagulation group (n = 5) are plotted. The identity of each designated metabolite **(a–i)** is shown in Supplementary Table. **(B)** The ratio of oxidized glutathione (GSSG) to reduced glutathione (GSH) in plasma samples collected before MRSA inoculation are normalized to 1, and the relative abundance of GSSG to GSH in plasma samples collected 12 h after MRSA inoculation with or without rTM treatment at 6-h group are plotted. *P < 0.05 as determined by Wilcoxon signed-rank test. **P < 0.05 as determined by Wilcoxon rank sum test.

Next, we analyzed metabolites in the plasma of MRSA-infected rats comprehensively to ascertain the potential effects of rTM administration. A total of 209 metabolites of known structures were identified and profiled. The relative abundance and *P*-value of each metabolite in the rTM treatment at 6-h group with reference to the no-anticoagulation group are shown in Fig. 4A, and the identity of each metabolite is shown in Supplementary Table. Among the metabolites, octanoylcarnitine levels were increased significantly in the rTM treatment at 6-h group compared with those in the no-anticoagulation group, whereas levels of oxidized glutathione, glycodeoxycholate and uridine 5′-diphosphate were decreased significantly (Supplementary Table). The relative abundance of oxidized glutathione to reduced glutathione was also decreased by rTM administration 6 h after MRSA infection (Fig. 4B). These findings suggested that rTM treatment may alleviate oxidative stress, bile-acid stasis, nucleotide accumulation, and recover fatty-acid metabolism.

## Discussion

Recent studies have raised fears that anticoagulant therapy might dampen the protective role of immunothrombosis and, thus, might worsen infectious diseases. Using a rat model of bloodstream infection with MRSA, we found that treatment with rTM neither increased bacterial burden nor worsened overall survival if rTM was used after the infection. Instead, rTM treatment alleviated oxidative stress, as evidenced by the decrease in the level of oxidized glutathione.

Bacterial passage from one compartment to another (e.g., from the intravascular space to the extravascular space) was facilitated by rTM treatment in our study. This finding might not be specific to rTM but common to various anticoagulants, including hirudin^8^ and heparin (data not shown). However, the increase of bacteria in the extravascular space seemed to be accompanied by a decrease of bacteria in the intravascular space, and so the bacterial burden in the whole organ was little-affected by rTM treatment. Considering that rTM did not worsen the survival rate of MRSA-infected rats if used after the infection, rTM treatment might not dampen the protective role of immunothrombosis.

Metabolomics analysis revealed that the increase in the level of oxidized glutathione in response to MRSA infection was alleviated by rTM administration 6 h after MRSA infection. Glutathione plays a major part in cellular defenses against oxidative stress^19^. In exchange for the reduction of target proteins, glutathione (also known as reduced glutathione or GSH) is converted to glutathione disulfide (also known as oxidized glutathione or GSSG). The GSSG:GSH ratio is thought to be a marker of cellular toxicity and is associated with mortality in septic-shock patients^20^. In the present study, the relative abundance of GSSG to GSH was increased in response to MRSA infection, and was significantly decreased by rTM administration 6 h after MRSA infection. These findings suggest that rTM alleviates oxidative stress, possibly through the maintenance of vascular patency or its intrinsic antioxidant properties^21^.

The timing of anticoagulation might also be controversial. Anticoagulant drugs have been administered before infection in studies for immunothrombosis^8,11^ whereas rTM has been administered after infection onset in clinical settings^22^. We administered rTM immediately before or 6 h after MRSA infection, and found that rTM administration 6 h after MRSA infection might be better in terms of improved survival. Although the best timing for clinical anticoagulation was not specified in our study, signs of organ dysfunction in addition to evident DIC might be important. Further studies are needed to gain insight into the appropriate timing for anticoagulation during sepsis treatment.

Our study had several limitations. First, it is not clear whether our findings in rats can be transferred to humans. Although studies have shown that rTM is effective in mitigating DIC in rats and humans^22,23^, activated protein C-dependent actions might not be fully expected in rats, unlike humans^24^. Second, it is not clear whether our findings are applicable to infection models other than bloodstream infection with MRSA. Third, observation beyond 18 h was not possible in this study because rats became seriously ill at about this point. Fourth, the number of rats in each group was low due in part to the complexity of experiments using rats with jugular-vein catheterization and live bacteria. Fifth, erroneous inferences may have occurred when multiple comparisons were conducted simultaneously in metabolomics analysis. Further studies are necessary to confirm the findings of our study.

## Conclusions

rTM did not promote bacterial propagation but alleviated oxidative stress in our rat model of bloodstream infection with MRSA. Further large-scale studies are needed to confirm these findings.

## Supplementary information

Supplementary Table.

## Data Availability

The datasets used and/or analyzed during the current study are available from the corresponding author on reasonable request.

## Abbreviations

CFU: Colony-forming unit
DIC: Disseminated intravascular coagulation
GSH: Reduced glutathione
GSSG: Oxidized glutathione
MRSA: Methicillin-resistant *Staphylococcus aureu*
NETs: Neutrophil extracellular traps
PBS: Phosphate buffered saline
rTM: Recombinant thrombomodulin
UDP: Uridine 5′-diphospate
