# Supplementary material for: Recombinant thrombomodulin alleviates oxidative stress without compromising host resistance to infection in rats infected with methicillin-resistant Staphylococcus aureus
Source: Sci Rep. 2020 Oct 15;10:17413. doi: 10.1038/s41598-020-74529-4 (PMC7566838; doi:10.1038/s41598-020-74529-4)
Supplement: Supplementary file 1 — Supplementary Table. [file 41598_2020_74529_MOESM1_ESM.docx]

**Supplementary Information**

**Recombinant thrombomodulin does not promote bacterial propagation but alleviates oxidative stress in rats infected with methicillin-resistant *Staphylococcus aureus***

Takashi Ito, MD, PhD^1,2^, Binita Shrestha^1,3^, PhD, Yasuyuki Kakihana, MD, PhD^2^, Ikuro Maruyama, MD, PhD^1^

^1^Department of Systems Biology in Thromboregulation, Kagoshima University Graduate School of Medical and Dental Sciences, Kagoshima, Japan

^2^Department of Emergency and Intensive Care Medicine, Kagoshima University Graduate School of Medical and Dental Sciences, Kagoshima, Japan

^3^Department of Medicinal Chemistry, Rogel Cancer Center, College of Pharmacy, University of Michigan, Ann Arbor, MI, USA

|  | **Metabolite** | **Group 2 vs. 1** | | **Group 4 vs. 2** | | **Group 4 vs. 3** | |
| --- | --- | --- | --- | --- | --- | --- | --- |
|  |  | Ratio | *P* value | Ratio | *P* value | Ratio | *P* value |
| a | L-Cys-glutathione disulfide | 8.24 | 0.116 | 0.08 | 0.104 | 0.07 | 0.331 |
| b | Oxidized glutathione | 4.39 | 0.051 | 0.30 | 0.048 | 0.22 | 0.333 |
| c | Galangin | 3.37 | 0.029 | 0.32 | 0.039 | 0.34 | 0.058 |
| d | Glycodeoxycholate | 4.73 | 0.002 | 0.53 | 0.016 | 0.85 | 0.673 |
| e | Palmitate (16:0) | 1.20 | 0.635 | 0.55 | 0.033 | 1.04 | 0.837 |
| f | UDP | 1.12 | 0.019 | 0.83 | 0.004 | 0.71 | 0.275 |
| g | Behenic acid (22:0) | 0.72 | 0.454 | 1.69 | 0.032 | 1.14 | 0.559 |
| h | Pseudouridine | 1.69 | 0.088 | 2.13 | 0.038 | 1.71 | 0.192 |
| i | Octanoylcarnitine (8:0) | 0.45 | 0.014 | 2.30 | 0.036 | 0.71 | 0.214 |

**Supplementary Table. Identity of designated metabolites (a-i) in Fig. 4A.**

The relative abundance and *P*-value of each metabolite are shown. Group 1: before MRSA inoculation (n = 5); group 2: 12 h after MRSA inoculation without anticoagulation (n = 5); group 3: 12 h after MRSA inoculation with rTM pretreatment (n = 4); group 4: 12 h after MRSA inoculation with rTM treatment at 6 h (n = 5). For comparison of two independent groups, the Student’s t-test was used.
